# Opioid Analgesic as a Positive Allosteric Modulator of Acid-Sensing Ion Channels

**DOI:** 10.3390/ijms25031413

**Published:** 2024-01-24

**Authors:** Dmitry I. Osmakov, Lyudmila V. Onoprienko, Aleksandr P. Kalinovskii, Sergey G. Koshelev, Vasiliy N. Stepanenko, Yaroslav A. Andreev, Sergey A. Kozlov

**Affiliations:** 1 Shemyakin—Ovchinnikov Institute of Bioorganic Chemistry, Russian Academy of Sciences, Ul. Miklukho-Maklaya 16/10, 117997 Moscow, Russia; osmadim@gmail.com (D.I.O.); onolv@mail.ru (L.V.O.); sknew@yandex.ru (S.G.K.); aya@ibch.ru (Y.A.A.); 2 Institute of Molecular Medicine, Sechenov First Moscow State Medical University, Trubetskaya Str. 8, Bld. 2, 119991 Moscow, Russia

**Keywords:** opioid peptide, acid-sensing ion channels, electrophysiology, positive allosteric modulation, ASIC pharmacology, analgesic peptide

## Abstract

Tafalgin (Taf) is a tetrapeptide opioid used in clinical practice in Russia as an analgesic drug for subcutaneous administration as a solution (4 mg/mL; concentration of 9 mM). We found that the acid-sensing ion channels (ASICs) are another molecular target for this molecule. ASICs are proton-gated sodium channels that mediate nociception in the peripheral nervous system and contribute to fear and learning in the central nervous system. Using electrophysiological methods, we demonstrated that Taf could increase the integral current through heterologically expressed ASIC with half-maximal effective concentration values of 0.09 mM and 0.3 mM for rat and human ASIC3, respectively, and 1 mM for ASIC1a. The molecular mechanism of Taf action was shown to be binding to the channel in the resting state and slowing down the rate of desensitization. Taf did not compete for binding sites with both protons and ASIC3 antagonists, such as APETx2 and amiloride (Ami). Moreover, Taf and Ami together caused an unusual synergistic effect, which was manifested itself as the development of a pronounced second desensitizing component. Thus, the ability of Taf to act as a positive allosteric modulator of these channels could potentially cause promiscuous effects in clinical practice. This fact must be considered in patients’ treatment.

## 1. Introduction

The development of opioid analgesics (OAs) is the most popular way to alleviate pathological pain in humans and animals. The most well-known OAs are such structurally related natural alkaloids as morphine, codeine, and thebaine; semi-synthetic and synthetic compounds such as diamorphine, oxycodone, fentanyl, etc. [1]. The mechanism of their action is the activation of opioid receptors (µ-, κ-, and δ-opioid receptors), which leads to the hyperpolarization of the pre- or postsynaptic membrane and, consequently, to a decrease in the nociceptive signaling [2]. The synapse contains many types of receptors and ion channels involved in the process of membrane depolarization that can be influenced by the hyperpolarizing effect of OAs [3,4]. The set of these channels includes acid-sensing ion channels (ASICs).

ASICs are proton-gated sodium channels that are predominantly expressed on the postsynaptic membrane of cells of the central and peripheral nervous systems (CNS and PNS), as well as in the glia, the retina, the pulmonary epithelium, and the immune cells [5]. Four genes encode six isoforms of ASICs (ASIC1a, 1b, 2a, 2b, 3, and 4), but only isoforms 1a, 1b, 2a, and 3 form functional homomeric channels [6,7]. Normally, a fast and strong acidic stimulus evokes ASICs’ opening, followed by their rapid desensitization, whereas a moderate acidity(conditioning pH) leads to a steady-state desensitization (SSD), which is expressed as a decrease in an open channel’s probability for a strong acidic stimulus [8,9]. Acidification during the release of vesicles by synaptic transmission and high ASICs content on the postsynaptic membrane suggests that ASIC channels are one of the potent participants in the transmission of nociceptive signals [10,11,12].

Today, compounds with activity both toward opioid receptors and ASIC channels are known. Thus, in experiments on the rat dorsal root ganglion (DRG) neurons, opioid receptor ligands, such as oxycodone, fentanyl, and endogenous endomorphin peptides, had a positive modulating effect directly on the ASIC channels [13,14], while morphine and DAMGO acted as negative modulators on the ASIC channels, which were mediated by the μ-opioid receptor and cAMP-dependent signal pathway [15]. It has also been shown in experiments in vitro and ex vivo that the dynorphin endogenous opioid peptides can restore the proton-evoked currents of ASIC1a and ASIC1a-containing channels at a weakly acidic conditioning pH, i.e., protect them from SSD [16,17,18].

Here, we studied the mechanism of action of the synthetic opioid peptide tafalgin (Taf) on ASIC channels. Taf is a tetrapeptide (Tyr-D-Ala-Phe-Gly-NH_2_) that has been shown to selectively activate μ1-opioid receptors. In this regard, after preclinical and clinical trials, this peptide was approved for use in clinical practice in Russia as an analgesic drug for subcutaneous administration (single dose was 4 mg/mL) [19]. We found that Taf is a positive allosteric modulator of ASIC1a, ASIC3, and ASIC3-containing heteromeric channels.

## 2. Results

### 2.1. The Action of Tafalgin on Homomeric ASIC Channels

**Figure 1:**
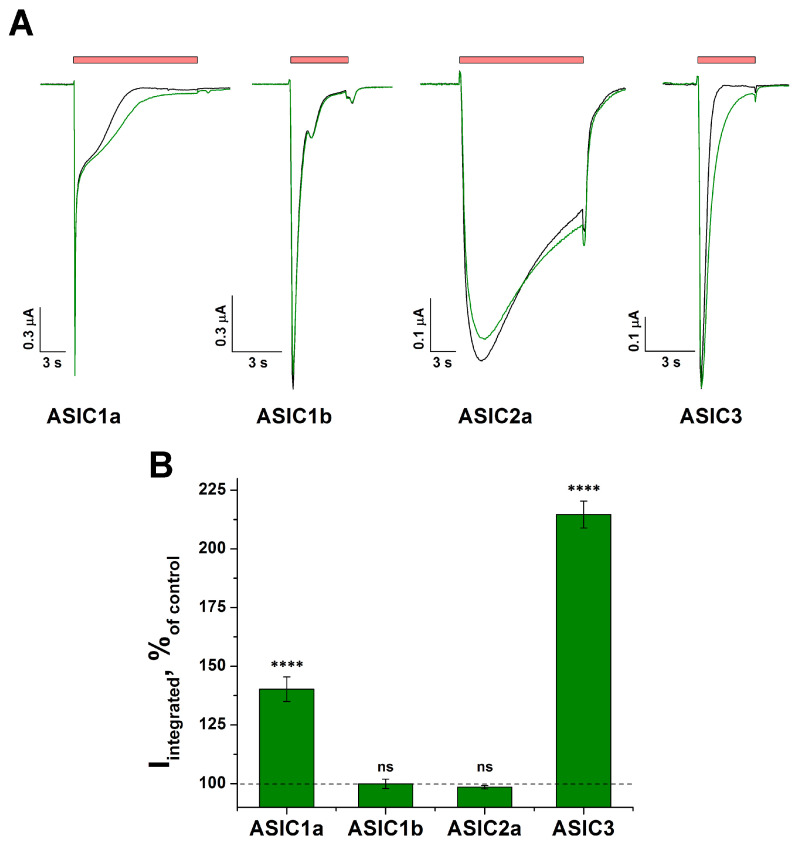
Effect of tafalgin (Taf) on the rat homomeric ASIC isoforms. (**A**) Representative current traces demonstrating the effect of 0.3 mM Taf (green line) on channels’ response to the pH 5.5 activating stimulus (red bar). (**B**) Bar plot of the integral current expressed as a percentage of the corresponding control currents (*n* = 5). The holding potential (V_h_) was −50 mV. Data are presented as mean ± SEM; ns, non-significant, **** *p* < 0.001 vs. control, unpaired *t*-test.

The testing of tafalgin (Taf) was carried out using the two-electrode voltage clamp method on the *Xenopus laevis* oocytes that heterologously expressed rat homomeric ASIC1a, 1b, 2a, and 3 channels. The action of Taf was assessed by the integral current. Taf (0.3 mM) exhibited a modulating effect on the ASIC1a and ASIC3 channels, which was expressed as a significant increase in the integral current by 40 ± 5% (*p* < 0.001) and 115 ± 6% (*p* < 0.001), respectively (Figure 1A,B). No effect was found on the ASIC1b isoform (*p* = 0.09), and a non-significant effect was observed on ASIC2a (*p* = 0.06) (Figure 1A,B).

### 2.2. The Mechanism of the Tafalgin Action on the Rat ASIC3 Channel

**Figure 2:**
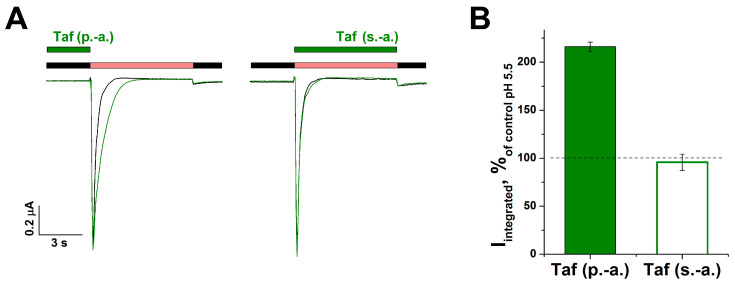

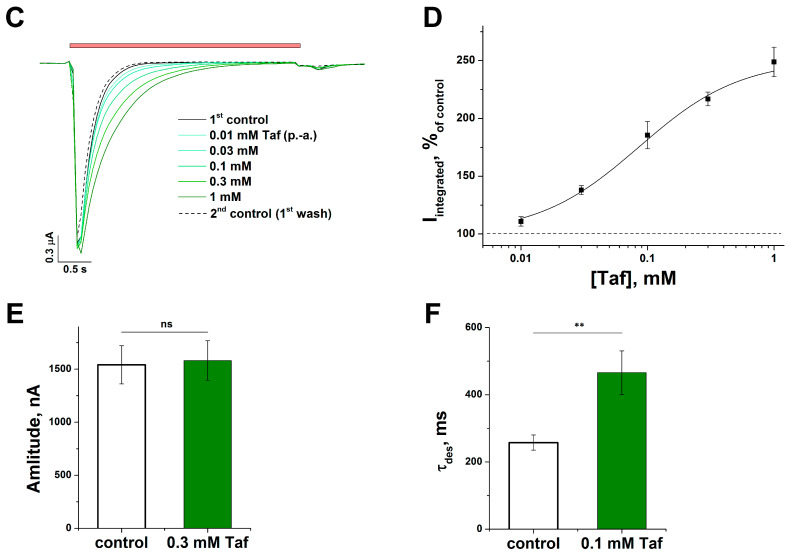
Effect of tafalgin (Taf) on the rat ASIC3 channels. (**A**) Representative unnormalized traces for a single cell, and (**B**) bar plot of the integral current for 0.3 mM Taf (green line and green bar) pre-applied for 30 s (p.-a.) and applied simultaneously (s.-a.) with the pH 5.5 stimulus (red bar) (*n* = 5). Black bar depicts conditioning pH 7.4. (**C**) Representative traces obtained from the same cell without (black solid and dotted lines) and with (green lines) a 30 s pre-application of Taf at various concentrations. Red bar depicts pH 5.5 stimulus. (**D**) Dose–response curve for the potentiating effect of Taf, obtained using the protocol in panel (**C**). Each point represents data from 9 cells. (**E**,**F**) Effect of Taf on the amplitude (**E**) and the time of exponential decay (τ_des_) (**F**) of the current (*n* = 9). Currents were induced by pH 5.5; conditioning pH was 7.4. Data are presented as mean ± SEM; ns, non-significant, ** *p* < 0.01, unpaired *t*-test.

The peptide had the most prominent effect on the rat ASIC3 channels (rASIC3), so the mechanism of its action was studied on this isoform. Two different protocols were applied to find out the Taf specificity to a closed or open channel state: incubation of the peptide with the channel in the resting state (protocol “p.-a.”) and simultaneous application of the peptide together with an acidic stimulus (protocol “s.-a.”). Taf (0.3 mM) produced a modulating effect only in “p.-a.”, increasing the integral current by 116 ± 5% (*p* < 0.001), whereas no effect was observed in the “s.-a.” protocol (*p* = 0.73) (Figure 2A,B). Thus, we could suggest the positive modulation of ASICs through the binding of Taf to the closed state of the channel.

The modulating effect depended on the concentration of Taf that was applied in the range of 0.01–1 mM (Figure 2C). The integral current values were determined by the Hill equation, where the half-maximal effective concentration (EC_50_) was 0.086 ± 0.024 mM, the Hill coefficient (n_H_) was 1.08 ± 0.16, and the maximum current (I_max_) was 251 ± 15% to control (pH drop from 7.4 to 5.5) (Figure 2D). It is worth noting that the current amplitude did not change (*p* = 0.9) (Figure 2E), while the rate of desensitization (τ_des_) increased significantly by 1.78 ± 0.12 times (258 ± 23 ms for control vs. 466 ± 65 ms for 0.1 mM Taf (*p* = 0.008)) (Figure 2F). In other words, the positive modulation occurred due to a decrease in the rate of desensitization (or an increase in the closure time) of the channel.

**Figure 3:**
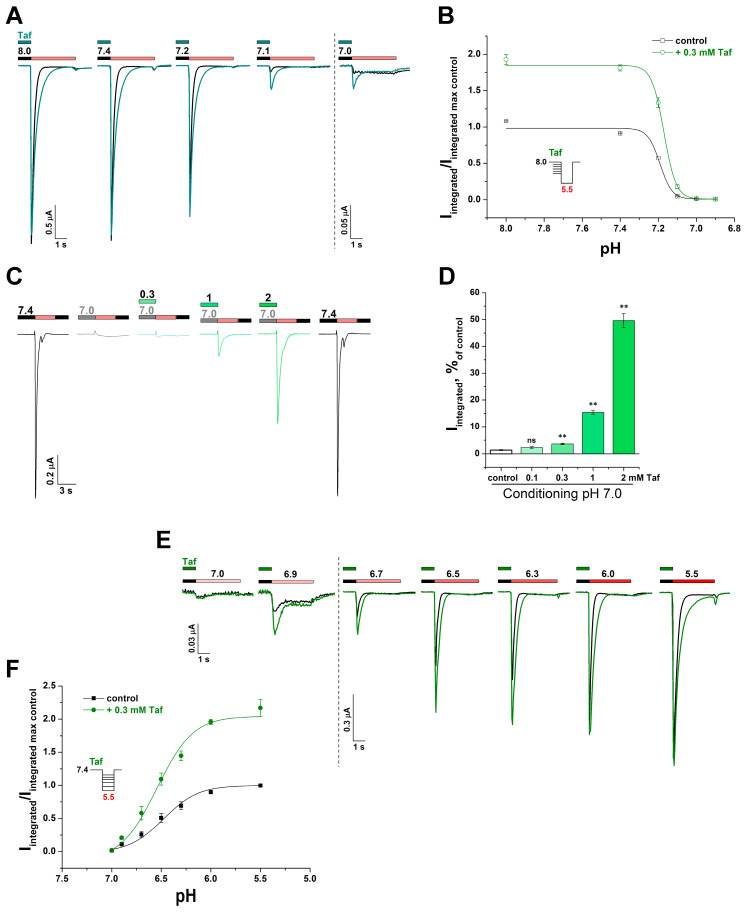
Taf influence on the rASIC3 pH dependence of the activation and the steady-state desensitization (SSD). (**A**) Representative current traces obtained from the same cell upon activation by a pH 5.5 stimulus (red bar) followed by pre-incubation with various conditioning pH either without (black line and black bar) or with 300 µM Taf (cyan line and cyan bar). (**B**) pH dependence of SSD either without or with 300 µM Taf (*n* = 5). (**C**) Representative current traces obtained from the same cell upon activation by a pH 5.5 stimulus (red bar) followed by pre-incubation with conditioning pH of 7.0 either without (gray line and grey bar) or with different Taf concentrations (green line and green bar). (**D**) Dependence of the integrated current to a pH 5.5 stimulus followed by a conditioning pH of 7.0 on the presence of Taf at various concentrations (*n* = 5). The response values are expressed as a percentage of the control value obtained at a conditioning pH of 7.4 and a stimulating pH of 5.5 (black lines on panel (**C**)). (**E**) Representative current traces obtained upon activation from conditioning pH of 7.4 (black bar) by various pH stimuli either without (black line) or with 300 µM Taf (green line and green bar). (**F**) pH dependence of activation with/without of 300 µM Taf (*n* = 9). Data are presented as mean ± SEM; ns, non-significant, ** *p* < 0.01 vs. control, Tukey’s test one-way ANOVA.

The peptide’s effect on the pH dependence of SSD and the activation of the channel were also examined. Taf (0.3 mM) did not lead to a significant change in either pH_SSD50_ (conditioning pH value, which causes a 50% decrease in the current) 7.19 ± 0.04 for control vs. 7.17 ± 0.01 for Taf or n_H_ (14.2 ± 5.9 vs. 13.6 ± 1.2) (*p* = 0.6) (Figure 3A,B). However, Taf had the ability to inhibit SSD. At a conditioning pH of 7.0, the integral current was 1.4 ± 0.2% of the control (pH drop from 7.4 to 5.5), while 0.3 mM Taf significantly increased the current to 3.6 ± 0.3% (*p* = 0.009), and 2 mM Taf increased the current to 50 ± 3% (Figure 3C,D).

Taf (0.3 mM) did not have a significant effect on pH_act50_ (stimulating pH value, causing a 50% increase in the current) 6.48 ± 0.09 for control vs. 6.55 ± 0.06 for Taf, and n_H_ of protons (2.5 ± 0.3 vs. 2.2 ± 0.2) (*p* = 0.5) (Figure 3E,F). Thus, Taf acted on the channel as a positive allosteric modulator and did not interfere with the interaction of protons with the channel.

### 2.3. Cooperative Action of Tafalgin with ASIC3 Channel Antagonists

**Figure 4:**
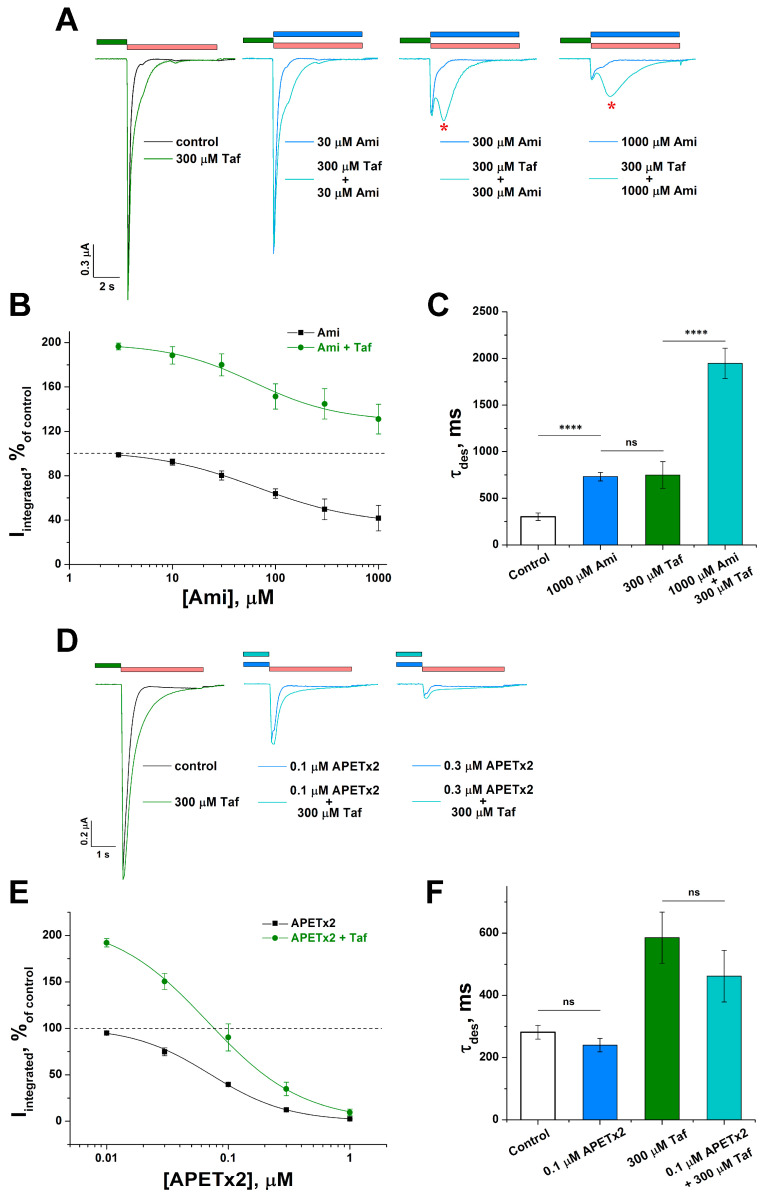
The combined action of Taf and the antagonists on rASIC3. (**A**,**D**) Representative traces of currents of rASIC3 alone (black line) and 30 s pre-incubation with 300 µM Taf (green line and green bar) in comparison with amiloride (Ami), applied simultaneously with the stimulus, (blue line and blue bar on (**A**)) and pre-incubated APETx2 (blue line and blue bar on (**D**)) or application of 300 µM Taf + corresponding antagonist (cyan line on (**A**,**D**)). Asterisk on the panel A depicts the second current component. (**B**,**E**) Dose–response inhibitory curves for Ami ((**B**), black line) or APETx2 ((**E**), black line) and for their combination with 300 µM Taf ((**B**,**E**), green line). Each point represents data from 5–6 cells. (**C**,**F**) Effect of 0.3 mM Taf alone and in the mixture with 1 mM Ami (**C**) and 0.1 µM APETx2 (**F**) on the rate of desensitization (τ_des_) (*n* = 5). In the case of Ami + Taf, τ_des_ was measured for the second component (marked with red asterisk in panel (**A**)). Currents were induced by pH 5.5 drop from conditioning pH of 7.4 (red bar). Data are presented as mean ± SEM; ns, non-significant, **** *p* < 0.001, Tukey’s test one-way ANOVA.

The well-known negative modulators of the ASIC3 channel in a competition experiment may help to localize the overlapped sites with the Taf binding site. We chose the low molecular weight compound amiloride (Ami) and the APETx2 peptide toxin as such negative modulators [20]. Ami inhibited the channel with calculated values of half-maximal inhibitory concentration (IC_50_) of 66.9 ± 5.2 µM (n_H_ of 0.87 ± 0.05) (Figure 4A,B), which was in a good agreement with the data published [21]. The inhibition was incomplete, and the residual integral current (lower asymptote in Figure 4B) was 36 ± 2% of the control. In the presence of the 0.3 mM Taf, the IC_50_ and n_H_ values were not significantly changed (61.5 ± 21.2 µM and 1.06 ± 0.37, respectively), but the residual current significantly increased up to 129 ± 9% (Figure 4B). The potentiating effect of both molecules exceeded the Taf effect as well. Therefore, we discovered an interesting synergistic effect realized via the development of the second current component (marked with a red asterisk in Figure 4A). The second component was especially pronounced at high Ami concentrations. The rate of desensitization of the second component was greatly slowed down, which was expressed as an increase in τ_des_ by 6.8 ± 0.9 times relative to the control (302 ± 40 ms for control vs. 1947 ± 162 ms for 0.3 mM Taf with 1 mM Ami) (Figure 4C). It was of interest that the solo application of 1 mM Ami or 0.3 mM Taf increased τ_des_ equally (up to 732 ± 46 ms for 1 mM Ami and 749 ± 143 ms for 0.3 mM Taf (*p* = 0.9)) and was significantly inferior to that of 0.3 mM Taf with 1 mM Ami (Figure 4C).

A different result was obtained for the combination of Taf and APETx2. APETx2 itself completely inhibited the channel with IC_50_ and n_H_ values of 71 ± 4 nM and 1.37 ± 0.05, respectively (Figure 4D,E), which was consistent with earlier reports [22,23]. In the presence of 0.3 mM Taf, the IC_50_ and n_H_ values were not changed significantly (68 ± 6 nM and 1.11 ± 0.05, respectively). Furthermore, no significant changes were observed for the rate of desensitization (τ_des_ 282 ± 22 ms for control vs. 240 ± 22 ms for 100 nM APETx2 (*p* = 0.2), τ_des_ 585 ± 82 ms for 0.3 mM Taf vs. 462 ± 83 ms for 0.3 mM Taf with 100 nM APETx2 (*p* = 0.3)) (Figure 4F).

### 2.4. Effect of Tafalgin on Human ASIC3 Channels

**Figure 5:**
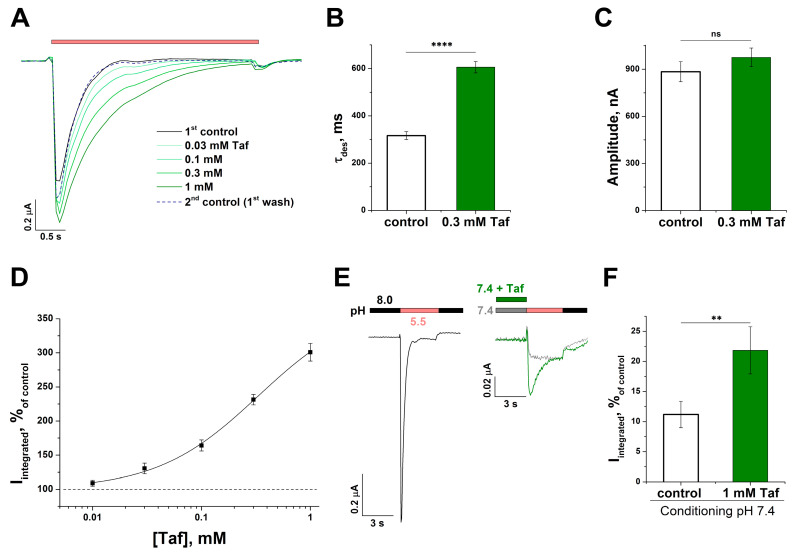
The effect of tafalgin (Taf) on human ASIC3 channels. (**A**) Representative unnormalized traces for a single cell without (black line) and with (green lines) a 30 s pre-application of Taf at different concentrations. Red bar depicts the pH 5.5 stimulus. (**B**,**C**) Effect of Taf on τ_des_ (**B**) and amplitude (**C**) of the current (*n* = 5). (**D**) Dose–response curve for the potentiating effect of Taf; each point represents data from 5 cells. Currents were induced by pH 5.5 drop from conditioning pH 8.0. (**E**,**F**) Representative current traces (**E**) and an integral current bar plot (**F**) for a single cell upon activation by a pH 5.5 stimulus followed by a conditioning pH of 7.4 either without (grey) or with 1 mM Taf (green) (*n* = 5). Response values are expressed as a percentage of the control (pH drop from 8.0 to 5.5, black line on panel (**E**)). Data are presented as mean ± SEM; ns, non-significant, ** *p* < 0.01, **** *p* < 0.001, unpaired *t*-test.

The effect of Taf on human ASIC3 channels (hASIC3) was also examined. Since it is known that hASIC3 are almost completely desensitized at a conditioning pH of 7.4 [24], in this case, Taf was incubated with the channel at a conditioning pH of 8.0. Taf had a concentration-dependent positive modulatory effect on hASIC3, similar to that on rASIC3. This effect was a consequence of a slowdown in the rate of desensitization, while the amplitude remained unchanged (τ_des_ 316 ± 16 ms for control vs. 606 ± 24 nA for 0.3 mM Taf (*p* < 0.001); the amplitude was 884 ± 64 nA for control vs. 975 ± 60 nA for 0.3 mM Taf (*p* = 0.3)) (Figure 5A–C). The calculated values for EC_50_ and n_H_ were 0.33 ± 0.08 mM and 0.94 ± 0.08, respectively, and I_max_ (upper asymptote) was determined as 373 ± 28% of the control (Figure 5D). Therefore, Taf exhibited an approximately 1.5-times-stronger effect on hASIC3, and shared the same cooperativity with both orthologs, but acted on rASIC3 at lower concentrations.

Taf also acted on the SSD of hASIC3. As mentioned above, hASIC3 loses the ability to generate the transient component of the current at a conditioning pH of 7.4, and the total integral current in this case was 11 ± 2% of that at a conditioning pH of 8.0. However, in the presence of Taf, the transient component was partially restored, and the total integrated current significantly increased up to 22 ± 4% (*p* = 0.005) (Figure 5E,F).

### 2.5. Effect of Tafalgin on ASIC1a Channels

**Figure 6:**
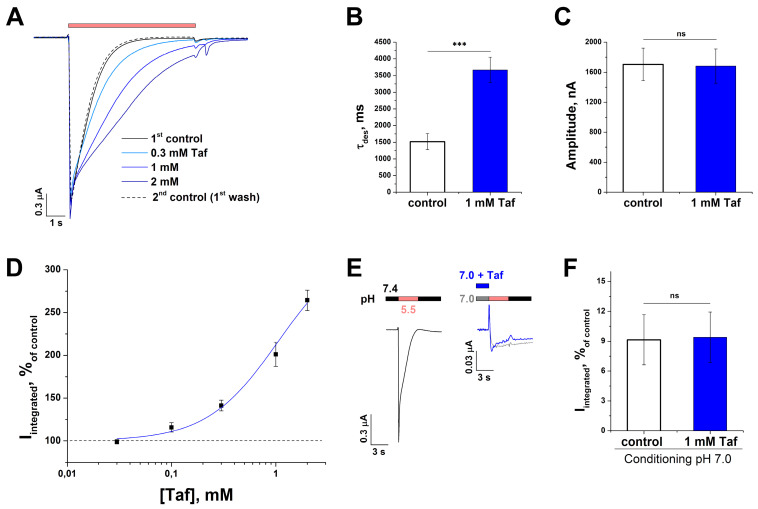
The effect of tafalgin (Taf) on rat ASIC1a channels. (**A**) Representative unnormalized traces for a single cell without (solid and dotted black line) and with (blue lines) a 30 s pre-incubation of Taf at different concentrations. Red bar depicts pH 5.5 stimulus. (**B**,**C**) Effect of Taf on τ_des_ (**B**) and amplitude (**C**) of the current (*n* = 5). (**D**) Dose–response curve for the potentiating effect of Taf (*n* = 10). The current was induced by pH 5.5 drop from conditioning pH of 7.4. (**E**,**F**) Representative current traces (**E**) and an integral current bar plot (**F**) for a single cell upon activation by a pH 5.5 stimulus followed by conditioning pH of 7.0 either without (grey) or with 1 mM Taf (blue) (*n* = 5). The response values are expressed as a percentage of the control (pH drop from 7.4 to 5.5, black line on panel (**E**)). Data are presented as mean ± SEM; ns, non-significant, *** *p* < 0.005, unpaired *t*-test.

Taf acted as a positive allosteric modulator on the rat ASIC1a channel (rASIC1a), although it was less potent than on rASIC3 (Figure 1A,B). The modulating effect of the peptide was concentration-dependent and manifested itself as the decrease in the rate of desensitization without amplitude alteration (τ_des_ 1520 ± 242 ms for control (pH drop from 7.4 to 5.5) vs. 3670 ± 381 nA for 1 mM Taf (*p* = 0.001); amplitude was 1705 ± 217 nA for control vs. 1682 ± 228 nA for 1 mM Taf (*p* = 0.9)) (Figure 6A–C). The calculated values for EC_50_ and n_H_ were 1.05 ± 0.27 mM and 1.3 ± 0.4, respectively, and the integral current I_max_ was 332 ± 103% of the control (Figure 6D). Thus, the affinity of Taf for rASIC1a was one order of magnitude lower than that for rASIC3, but the maximal integral current (I_max_) was the same (*p* = 0.45). In contrast to rASIC3, Taf showed no effect on the SSD of rASIC1a. At a conditioning pH of 7.0, the presence of 1 mM Taf retained the integral current at the control (pH drop from 7.0 to 5.5) current level (9.1 ± 2.5% for control vs. 9.4 ± 2.5% for 1 mM Taf (*p* = 0.9)) (Figure 6E,F).

### 2.6. Effect of Tafalgin on Heteromeric Rat ASIC Channels

**Figure 7:**
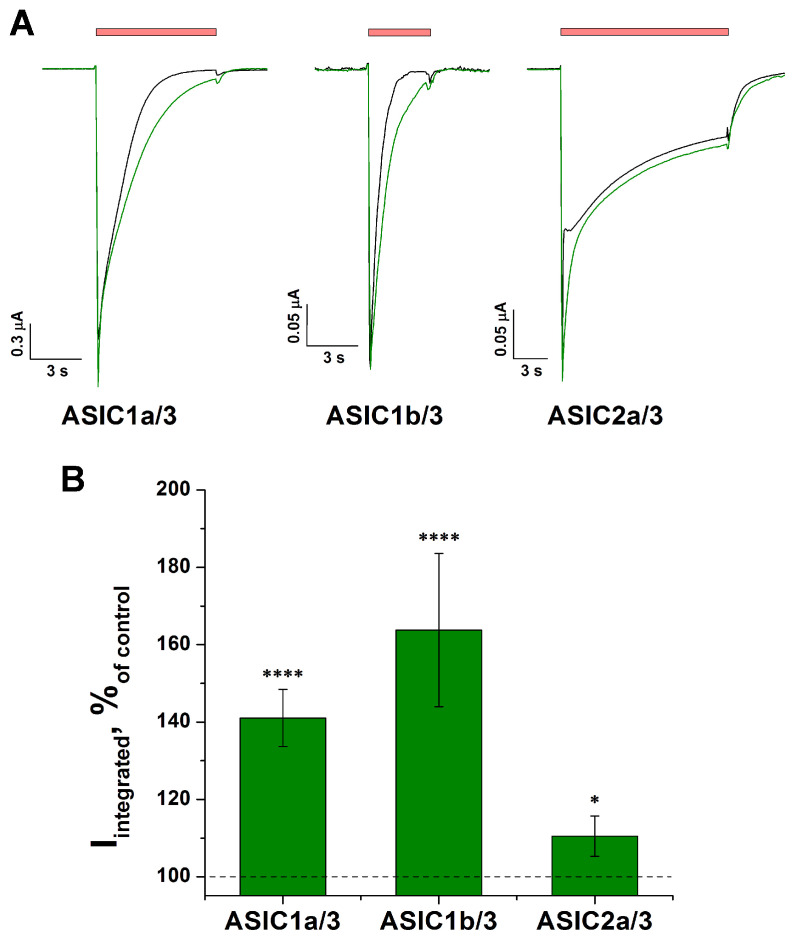
The effect of Taf on the heteromeric ASIC channels. Representative current traces (**A**) and bar plot (**B**), for the modulating effect of 0.3 mM Taf (green line) on current evoked by pH drop from 7.4 to 5.5 (red bar). The data in panel (**B**) are presented as the integral current, expressed as a percentage of control currents (black line) (*n* = 5). The data are presented as the mean ± SEM; * *p* < 0.05, **** *p* < 0.001 vs. control, unpaired *t*-test.

As was demonstrated above (Section 2.1), Taf had the strongest effect on ASIC3, whereas its effect on ASIC1b and ASIC2a was absent or insignificant. Therefore, we investigated how Taf acted on different heteromeric channels consisting of isoform 3 and another isoform: 1a, 1b, or 2a. A modulating effect of 0.3 mM Taf was observed for all three combinations (i.e., ASIC1a/3, 1b/3, and 2a/3 channels) (Figure 7A,B). Heteromeric ASIC1a/3 demonstrated an increase in current of 41 ± 7%, which absolutely coincided with the effect of Taf on the homomeric ASIC1a channels (Figure 1). The effect on the ASIC2a/3 heteromers was small but significant (increase in current by 10 ± 5% (*p* = 0.04)). Surprisingly, Taf had a strong modulating effect on ASIC1b/3, increasing the integral current by 64 ± 20%.

## 3. Discussion

In this study, we have investigated the pharmacological effect of tafalgin (Taf) on ASICs. We have demonstrated that Taf positively modulates the homomeric ASIC1a and ASIC3, as well as the ASIC3-containing heteromeric channels. These channels, preferably having a neuronal localization, play an important role in the functioning of CNS and PNS, and are involved in the regulation of synaptic plasticity and learning [25], the fear perception [26,27], and responses to ischemic stroke [28], multiple sclerosis [29], and nociception [12,30]. Positive modulators of ASICs are shown to have negative effects in pathological processes. For example, the coral snake toxin MitTx and the 2-guanidine-4-methylquinazoline synthetic low molecular compound were able to induce nocifensive behavior in mice through activation of ASIC1 and ASIC3 channels, respectively [31,32]. The well-known opioid peptides: dynorphins, which modulate the ASIC1, and RPRFa peptide from the *Conus* venom, which potentiates ASIC3, have also been shown to increase the acidosis-induced neuronal death and the pain sensitivity, respectively [33,34].

The *X. laevis* oocytes are usually surrounded by a thick outer membrane (i.e., vitelline membrane or jelly coat). This coat may have a significant effect on the magnitude of the current, the rate of desensitization, or the concentration–response relationship. Therefore, there is a possibility that the effect of the ligand on ASICs may be underestimated. Moreover, different levels of acidity in the bath solution used to induce ASIC currents may affect the thick membranes around the *Xenopus* oocytes, thereby affecting their physical properties. Thus, the vitelline membrane of the *Xenopus* oocytes used was removed completely (using collagenase and microtweezers; see Materials and Methods for details) to allow access to the cell for various manipulations.

Due to the modulating effect of Taf towards ASIC3 in peripheral neurons, the proven analgesic effect of Taf on human may be accompanied by the development of side effects such as pain, mechanical hyperalgesia, inflammatory muscle and joint hyperalgesia, etc., where ASIC3 plays a crucial role [35,36]. In addition, the significant modulating effect on ASIC1b/3 heteromers (Figure 7) may also have a negative impact on both peripheral acute and acid-induced chronic pain, where the ASIC1b-containing channels also play an important role [37,38]. Indeed, the recommended dose for subcutaneous administration is a ~9 mM solution of Taf, which is approximately 30 times higher than the EC_50_ for the modulation of human ASIC3 channels. Thus, numerous side effects stated on the manufacturer’s website for “Medicine Tafalgin, solution for subcutaneous administration” (https://sotex.ru/production/catalog/detail.php?ELEMENT_ID=4952 (accessed on 22 January 2024)), such as peripheral neuropathy, itching, and pain at the injection site, may be a consequence of the positive modulation of ASIC channels. The concentration of Taf that increases the susceptibility of cells to acidification significantly exceeds the concentration required for µ-opioid activation; therefore, only local adverse effects are possible.

Taf is structurally close to the endogenous peptides endomorphins, which are agonists of µ-opioid receptors. Earlier, the ability to positively modulate ASIC3 channels to a greater extent and ASIC1a channels to a lesser extent has been demonstrated for endomorphins [13]. However, the mechanisms of action of Taf and endomorphins on ASIC3 differ significantly. Firstly, endomorphins positively modulate the non-desensitizing sustained component of the current, while Taf significantly slows down the desensitization rate of the transient component. Moreover, endomorphins cause an alkaline and acid shift in the pH-dependence of channel activation and desensitization, respectively [13], whereas Taf has no effect on the pH-dependence, demonstrating the properties of a positive allosteric modulator.

Oocytes, as a model heterologous expression system, allow the pure effect of the peptide on ASIC channels to be measured without any influence from opioid receptors (not yet expressed in cells). Thus, we were able to demonstrate the direct interaction between the Taf and ASIC channels. Unfortunately, the Taf’s binding site to the ASIC channel was not determined yet. However, based on the lack of competition between Taf and amiloride or APETx2 (Figure 4B,E), we assume this site location to be outside the binding sites for amiloride and APETx2 on the extracellular part of the ASIC channel.

Amiloride (Ami) is known as a dual-action ligand, capable of blocking ASIC channels and acting as an ASIC3-positive modulator [39]. Ami is able to reduce chronic morphine tolerance by suppressing morphine-induced microglial activation through ASIC3 inhibition [40]. Ami is also used in clinical practice as a potassium-sparing diuretic for the treatment of hypertension and heart failure. In our study, we combined both agents (Taf and Ami) and found the intriguing and unusual synergistic phenomena. One of the distinctive features of ASIC3 channels is the development of a sustained, non-desensitizing current component that follows the transient component and lasts throughout the entire moderately acidic stimulus [41]. However, in the presence of Taf and Ami (especially high concentrations of the latter), the transient component decreased, but a strongly pronounced second component appeared. This component was also desensitized like the transient component, although at a much lower rate. As far as we know, this phenomenon is described for the first time for the ASIC3 isoform. Since both remedies could potentially be prescribed to a patient, the combined effect on ASIC channels could lead to unpredictable consequences.

Thus, the positive modulation of ASICs by high doses of Taf, especially in combination with other drugs, may cause possible side effects or reduce the effectiveness of this therapeutic. This fact must be considered when developing a patient’s treatment plan.

## 4. Materials and Methods

### 4.1. Synthesis and Purification of Tafalgin

The tetrapeptide was prepared by manual solid-phase peptide synthesis on the Rink-amide polymer (100–200 mesh, load of 0.37 mmol NH_2_/g of the polymer, Novabiochem, Germany; the polymer amount was 0.99 g) using the Fmoc/Bu^t^ strategy. The following amino acid derivatives were used: Fmoc-Gly-OH, Fmoc-Phe-OH, and Fmoc-Tyr(Bu^t^)-OH (Iris Biotech, Marktredwitz, Germany), as well as Fmoc-*D*-Arg(Pbf)-OH (Novabiochem, Darmstadt, Germany) in the two fold excess relative to the content of amino groups of the polymer (0.37 mmol NH_2_/g polymer). The Fmoc group was removed by the double treatment with 40% 4-methylpiperidine in dimethylformamide (for 15 min and 10 min). The solid-phase synthesis included the following stages: 1. Removal of the Fmoc group; 2. Washing with dimethylformamide (8 mL × 10 mL); 3. Condensation with *N*,*N*’-diisopropylcarbodiimide (0.74 mmol; 2 equivalents relative to the content of the free amino groups of the polymer) in the presence of 1-hydroxybenzotriazole (0.89 mmol; 1.2 equivalents relative to *N*,*N*’-diisopropylcarbodiimide); and 4. Washing with dimethylformamide (3 mL × 10 mL). The completeness of the condensation reaction was confirmed by the bromophenol blue test. Upon the completion of the synthesis, the peptidyl polymer was washed with methylene chloride (3 mL × 15 mL) and dried in vacuum over KOH. The final deprotection with simultaneous cleavage of the peptide from the polymer was carried out by a two-step treatment. At first, the peptidyl polymer was suspended in 10% trifluoroacetic acid in methylene chloride and the suspension was transferred to a glass filter. The polymer was then slowly washed on the filter with a mixture of 5% trifluoroacetic acid and 1% triisopropylsilane in methylene chloride (3 mL × 10 mL), and treated with a mixture of trifluoroacetic acid, water, and triisopropylsilane (95%:2.5%:2.5% by volume) for 1.5 h. The resulting filtrate was combined with the first one and evaporated. The residue was dissolved in water, and subjected to a chromatographic purification on a Luna C_18_ column (10 mm × 250 mm; Phenomenex, Torrance, CA, USA). The purity of Tafalgin used in subsequent experiments was 97% (Figure S1).

### 4.2. Chemical Reagents and ASIC Ligands

Amiloride and salts for the buffer preparation were obtained from Sigma-Aldrich (St. Louis, MO, USA).The APETx2 peptide was produced with heterologous expression in the *Escherichia coli* as described [42]. Salts for the buffer preparation were obtained from ISOLAB Laborgeräte (Eschau, Bavaria, Germany).

Fresh working solutions of the compounds were prepared in ND96 buffer immediately before experiments, and the pH value of these solutions was controlled.

### 4.3. Xenopus laevis Oocyte Isolation and mRNA Injection

This study was performed in strict accordance with the World Health Organization’s International Guiding Principles for Biomedical Research Involving Animals. The protocol was approved by the Institutional Policy on the Use of Laboratory Animals of the Shemyakin–Ovchinnikov Institute of Bioorganic Chemistry of the Russian Academy of Sciences (Protocol Number: 351/2022; date of approval: 24 November 2022). All procedures were performed in accordancewith the guidelines of ARRIVE (Animal Research: Reporting of In Vivo Experiments) and the “European convention for the protection of vertebrate animals used for experimental and other scientific purposes” (Strasbourg, 18.III.1986). Female *X. laevis* frogs were kept at 20 ± 2 °C, and anesthetized with 0.17% tricaine methanesulfonate (MS222), after which a small part of the ovary was removed through an abdominal incision and placed in ND96 medium (96 mM NaCl, 2 mM KCl, 1.8 mM CaCl_2_, 1 mM MgCl_2_, and 5 mM HEPES titrated to pH 7.4 with NaOH). The animals operated on showed no signs of postoperative distress syndrome. For each animal, the interval between operations was at least three months. Connective tissue and follicular sheath elements were removed by treatment with collagenase (1 mg/mL in ND96 medium without calcium) for 2 h at room temperature. Then, healthy and mature oocytes were selected. If they had a vitelline membrane, an additional procedure for its removal was carried out using microtweezers (tip size 0.05 mm × 0.01 mm) (Dumont Tweezers #5 Straight Stainless Steel, Dumont, Switzerland). While oocyte was lightly fixed with one microtweezer, its vitelline membrane was pinched and pulled off by the second microtweezer.

Sorted healthy stage IV and V oocytes without any outer membranes were stored in an ND96 medium and 2–10 ng of cRNA were injected 16–18 h after the isolation. cRNAs synthesized from PCi plasmids containing the rat ASIC1a, 1b, 2a, and 3 isoforms, as well as from pVAX1 plasmid containing the human ASIC3, were injected into the oocytes using a Nanolitre 2000 microinjection system (World Precision Instruments, Sarasota, FL, USA). After the injection, the oocytes were kept for 2–3 days at 18 °C, and then for up to 7 days at 15 °C in an ND96 medium supplemented with gentamicin (50 μg/mL).

### 4.4. Two-Electrode Voltage-Clamp Recordings

Whole-cell ASICs currents were recorded using a two-electrode voltage clamp technique with a GeneClamp 500 amplifier (Axon Instruments, Union City, CA, USA) at a holding potential of –50 mV (Figure S2). It should be noted that the reversal potential of +22 mV is slightly different from the reversal potential of Na+, which may indicate interference from potassium and chloride channels. Microelectrodes were filled with 3 M KCl solution. The external bath solution was ND-96 (pH 7.4, 7.3, and 7.2) and ND96, in which 5 mM of HEPES was substituted for 5 mM HEPPS (pH 8.0) or for 5 mM MOPS (pH 7.0, 6.9). The activating solution was ND96 in which 5 mM HEPES was replaced by 10 mM MOPS (followed by the pH adjusting to 7.0, 6.9, and 6.7), or 10 mM MES (followed by the pH adjusting to 6.5, 6.3, 6.0, and 5.5). The flow rate and fast solutions exchange in the registration chamber were controlled by an in-house computer valve system. The data were filtered at a frequency of 10 Hz and digitized at a frequency of 100 Hz using an L780M ADC (LCard, Moscow, Russia).

### 4.5. Data Analysis

The data were analyzed using OriginPro 8.6.0 (OriginLab, Washington, DC, USA). Dose–response data for potentiating and inhibitory actions of ligands were fitted with the Hill equation Ix = I_0_/[1 + ([x_0_]/[x])^n_H_], where I_x_ was the ionic integral current at a given concentration of ligand [x], I_0_ was the ionic current in the absence of ligand, x_0_ was the concentration at which a ligand exhibited half of its maximal effect, and n_H_ was the Hill coefficient.

The pH–response dependence of the current was fitted by the Hill equation F_1_(x) = ((A1 − A2)/(1 + (x/[H+]_50_)^n_H_)) + A2; where [H+]_50_ was the concentration of protons at which the half-maximal integral current was reached, A1 was the minimum response value, A2 was the maximum response value, and nH was the Hill coefficient. The maximum value (I_max_) was calculated from the integral values obtained at each pH for each cell by the individual fitting. The data were then normalized to the calculated I_max_ value. The normalized data were averaged and fitted to the logistic equation F_1_(x).

The rate of curve decay was fitted for each individual experiment using a single exponential decay, F_2_(x) = A*e^(−x/τ_des_) + A_0_, where A_0_ was the minimal current response, and τ_des_ was the decay constant.

All data are presented as the mean ± SEM. The differences between groups were tested with an unpaired *t*-test and a one-way analysis of variance (ANOVA), followed by the Tukey’s post hoc test. A difference of *p* < 0.05 was considered to be significant.

## Data Availability

The data that support the findings of this study are available from the corresponding author upon reasonable request.

## Supplementary Materials

The following supporting information can be downloaded at: https://www.mdpi.com/article/10.3390/ijms25031413/s1.
