# Supplementary material for: Opioid Analgesic as a Positive Allosteric Modulator of Acid-Sensing Ion Channels
Source: Int J Mol Sci. 2024 Jan 24;25(3):1413. doi: 10.3390/ijms25031413 (PMC10855113; doi:10.3390/ijms25031413)
Supplement: Supplementary file 1 [file ijms-25-01413-s001.zip › ijms-2775924-supplementary.pdf]

Supplementary Materials

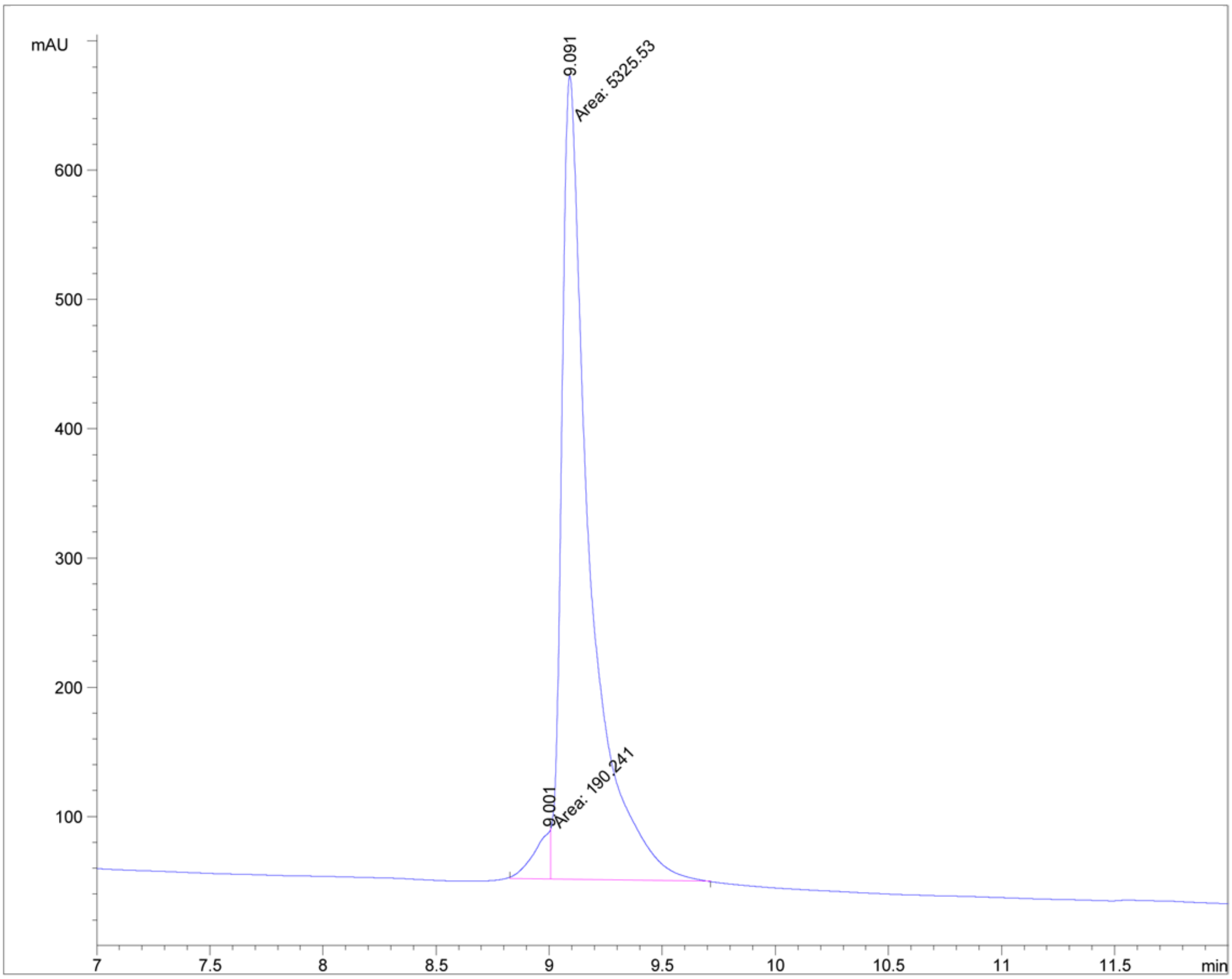

Figure S1. The purity of the tafalgin sample was analysed on Xbridge C18 column 130A (Waters, 4.6x250 mm).

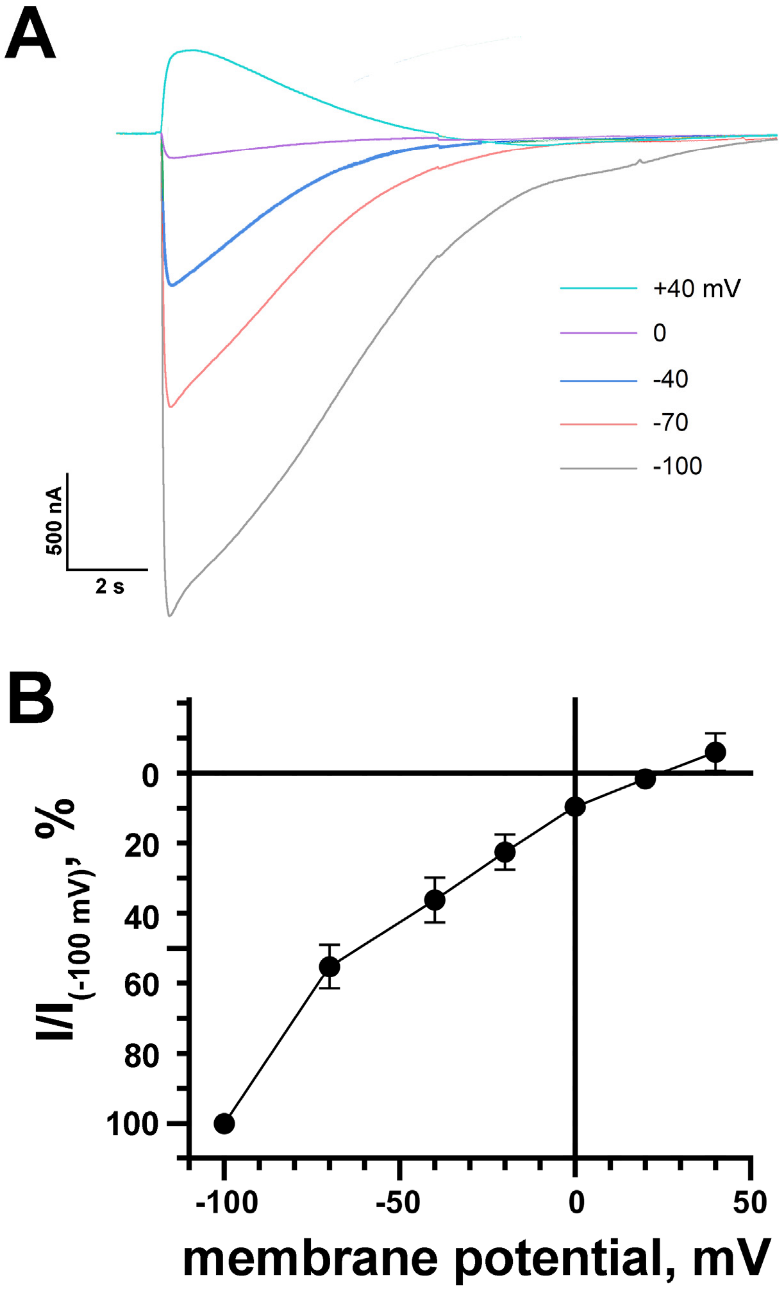

Figure S2. Current-voltage (I-V) relation of rat ASIC1a current. (A) Representative current traces at different holding potentials of ASIC1a heterologously expressed in *X. laevis* oocytes. (B) I-V plot of the currents recorded in the whole-cell configuration. For the I-V plot, currents elicited by pH 5.5 at different holding potentials were recorded. Current amplitudes were normalized to the amplitudes of the currents at V = -100 mV and plotted against the holding potentials. Each dot represents mean ± SEM (n = 5).
